# Evolutionary Consequence of a Trade-Off between Growth and Maintenance along with Ribosomal Damages

**DOI:** 10.1371/journal.pone.0135639

**Published:** 2015-08-20

**Authors:** Bei-Wen Ying, Tomoya Honda, Saburo Tsuru, Shigeto Seno, Hideo Matsuda, Yasuaki Kazuta, Tetsuya Yomo

**Affiliations:** 1 Faculty of Life and Environmental Sciences, University of Tsukuba, 1-1-1 Tennodai, Tsukuba, Ibaraki, 305–8572, Japan; 2 Section of Molecular Biology, Division of Biological Sciences, University of California San Diego, La Jolla, California, 92093, United States of America; 3 Graduate School of Information Science and Technology, Osaka University, 1–5 Yamadaoka, Suita, Osaka, 565–0871, Japan; 4 ERATO, JST, 1–5 Yamadaoka, Suita, Osaka, 565–0871, Japan; 5 Graduate School of Frontier Biosciences, Osaka University, 1–5 Yamadaoka, Suita, Osaka, 565–0871, Japan; University of Wisconsin-Madison, UNITED STATES

## Abstract

Microorganisms in nature are constantly subjected to a limited availability of resources and experience repeated starvation and nutrition. Therefore, microbial life may evolve for both growth fitness and sustainability. By contrast, experimental evolution, as a powerful approach to investigate microbial evolutionary strategies, often targets the increased growth fitness in controlled, steady-state conditions. Here, we address evolutionary changes balanced between growth and maintenance while taking nutritional fluctuations into account. We performed a 290-day-long evolution experiment with a histidine-requiring *Escherichia coli* strain that encountered repeated histidine-rich and histidine-starved conditions. The cells that experienced seven rounds of starvation and re-feed grew more sustainably under prolonged starvation but dramatically lost growth fitness under rich conditions. The improved sustainability arose from the evolved capability to use a trace amount of histidine for cell propagation. The reduced growth rate was attributed to mutations genetically disturbing the translation machinery, that is, the ribosome, ultimately slowing protein translation. This study provides the experimental demonstration of slow growth accompanied by an enhanced affinity to resources as an evolutionary adaptation to oscillated environments and verifies that it is possible to evolve for reduced growth fitness. Growth economics favored for population increase under extreme resource limitations is most likely a common survival strategy adopted by natural microbes.

## Introduction

Improved fitness of the cells surviving evolution is commonly evaluated by the growth rate in cell propagation, *i*.*e*., the rate at which population size increases. Rapid reproduction indicates high fitness as it allows cells to form large population sizes as a strategy against the individual cell death caused by environmental perturbations, and such populations are highly competent in occupying their habitats. Thus far, experimental demonstrations, by means of “evolution in action” within an observable time scale [1, 2], often provided the fast-growing cells as an evolutionary consequence and the experimental findings and theoretical insights have relied largely on this fast-growing property [3–8]. However, microorganisms found in nature quite often fall into “sleep-like” states such as dormancy [9–11] and the viable but nonculturable (VBNC) state [12–14]. These cellular physiologies were proposed as the survival strategies for the cells to maintain themselves, most likely at the minimum cost in extremely severe conditions [11, 15, 16]. These strategies indicate that fast-growing is not common in nature. The contrastive growth physiologies in the laboratory and in nature led us to consider whether it is possible to evolve experimentally a phenotype (cellular physiology) that is balanced between growth and maintenance to reflect a natural habitat.

Studies on the cells of slow or paused growth phases, such as the stationary phase [17] and of the growth under limited resource (hunger phase) [18] found different physiological characteristics from that of fast or exponentially growing cells. Bacterial cells experiencing prolonged stationary phases showed changes in either phenotypes or genotypes, such as reduced cell sizes [19, 20], decreased protein abundances [21] or RNA amounts [22], improved stress resistance [23], genetic loss [24] or heterogeneity [25]. In particular, a number of pioneer studies observed a phenotype of a growth advantage in stationary phase (GASP) [26, 27], as well as the survival trade-offs [28, 29]. These intriguing findings strongly suggested that the growth fitness and the tolerance to stress were conditionally balanced in cells, supported by the reports of microbial gene expression patterns exhibiting task divisions in the stress response and rapid growth [30, 31]. Therefore, microorganisms might have been evolved to tolerate severe environments as well as to optimize their growth [32].

Thus far, little is known about the evolutionary strategy by which microorganisms remain alive in both good and bad conditions. It is unclear what growth economics are adopted by the cells to optimize the balance between growth and maintenance in nature. Although experimental evolution is a powerful approach to investigate fitness determinants, few experiments have precisely targeted the oscillated stationary phase in bacterial batch cultures. Therefore, to understand the survival strategies in the fluctuated environments, we questioned what the growth physiology would become in a slowly and irregularly oscillated environment, similar to what occurs in nature, by means of experimental evolution.

To address the question, we performed a long-term evolution experiment involving starvation and re-feeding with a laboratory *Escherichia coli* strain, as microbes in nature continually cycle between good and bad conditions (*e*.*g*., hydration-dehydration, freeze-thaw and starvation-nutrition cycles) and resource limitation may be one of the most common stresses they experienced. The *Escherichia coli* cells that survived the 290-day long repeated starvation and resuscitation conditions adopted a considerably slow metabolism, similar to the changes exhibited by microbes in nature. The slowly oscillating culture environment led to a transition in cell physiology, from rapid propagation in rich conditions to high competence in poor conditions. This strategic transition was associated with ribosomal mutations, indicating that the improved sustainability was partially accomplished by the genetic fixation of mutations whose effect was to suppress resource-consuming translation machinery. The present study provides a good example of evolutionary consequences: growth economics for maintaining life should take into account both the speed of reproducing under favorable conditions and the capacity to survive in severe environments.

## Results

### Experimental evolution in a nutritional oscillated environment

**Fig 1:**
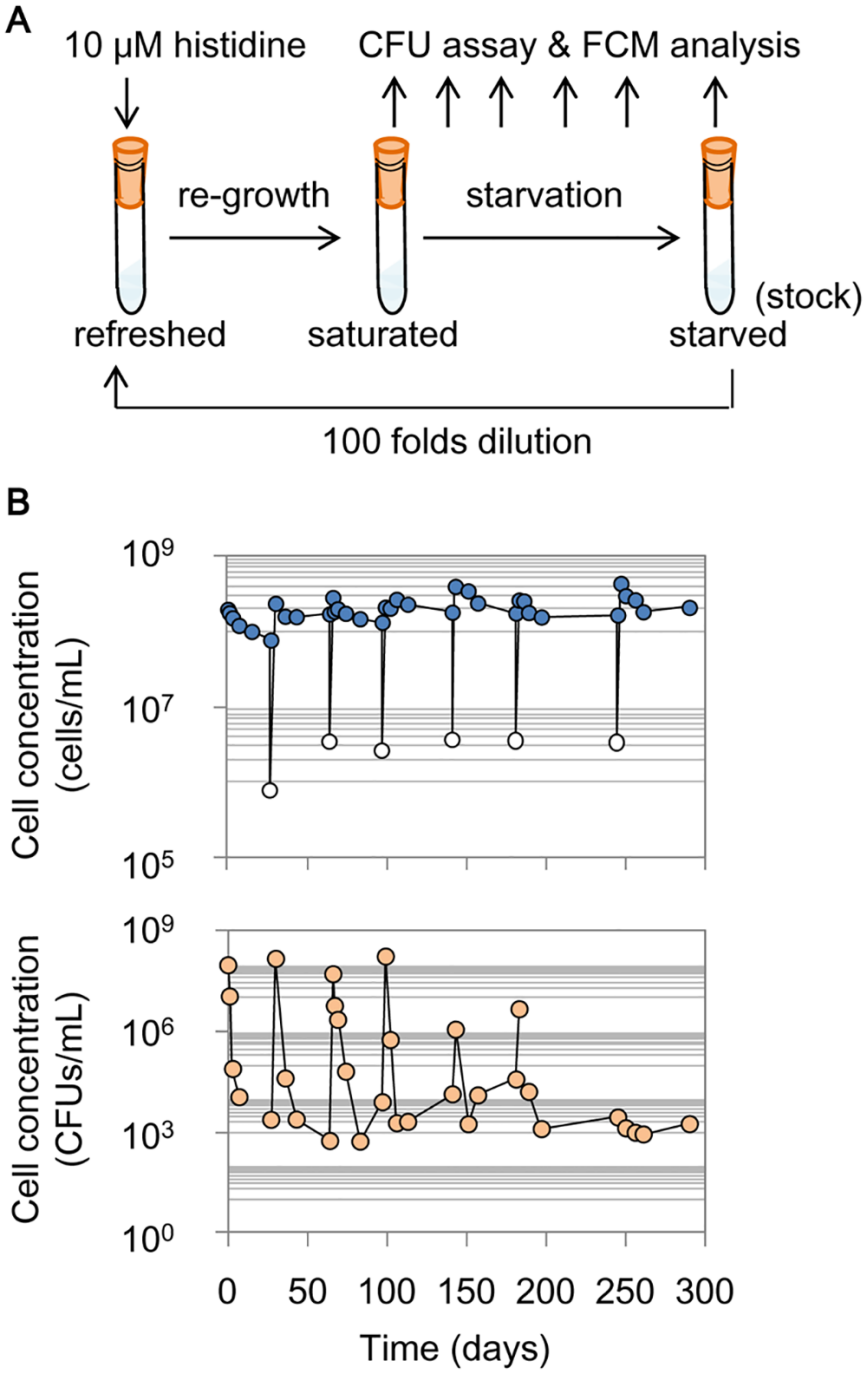
Repeated starvation and re-growth. **A.** Scheme of the evolution experiment. **B.** Temporal changes in the population density. The cell concentrations were counted by flow cytometry (FCM count, upper panel), and the active cells were detected with a colony formation unit (CFU) assay (bottom panel). The blue- and orange-filled circles represent the sampling points, and the white circles indicate the cell concentrations after 100-fold dilutions.

Long-term experimental evolution mimicking repeated starvation and resuscitation was performed in test tubes (Fig 1A). A laboratory *Escherichia coli* strain that required histidine for growth was used. The cells were grown in the presence of 10 μM histidine until saturation, in which the final cell concentrations were commonly ~3×10^8^ cells/mL. The saturated cell cultures were left in the same test tubes undergoing continuous incubation (Fig 1B), which served as the starvation phase, in which the cells were assumed to compete for maintenance. Resuscitation was initiated by transferring a portion of the starved populations at the endpoint to a fresh histidine-supplied medium (Fig 1B, upper panel, open circles). Cells in this growing (re-growth) phase were assumed to compete for growth fitness. The cell cultures were sampled during the repeated starvation and resuscitation; they were subjected to flow cytometry (FCM) and colony-forming unit (CFU) analyses at intervals varying from one day to one month. A total of seven rounds of starvation and resuscitation were performed (Fig 1, S1 Fig). The endpoint cell populations of each round of starvation were referred to as R1 to R7, and the ancestor was R0.

Temporal changes in the cell populations demonstrated the success of the cells in surviving the selection pressures on both growth and maintenance. FCM and CFU analyses showed equivalent saturated population sizes in response to the histidine supplement but a substantial dissimilarity under starvation (Fig 1B, S1 Fig), as the two methods detected different cell formats [33, 34]. In all three replicates, the number of CFU cells surviving starvation is kept rather constant around 10^2^−10^3^ cells/mL, which indicated the strength of competition in the resource-oscillated environments. Note that the roughly similar CFU counts on all three types of agar plates (S1 Fig) indicated that there was no contamination during the evolution experiment.

### Genome mutations fixed in the gene functions involved in translation and transport

One of the three lineages showing the most significant changes in growth fitness of the final evolved population (R7) was subjected to further analyses. Genome resequencing analysis was performed for the ancestor (R0) and the evolved population (R7). Detected mutations were confirmed by the Sanger method in all eight populations from R0 to R7. Repeated tests confirmed that ten mutations (Table 1) were completely fixed (approximately 100% frequency) in the final population, R7. How these mutations accumulated during the evolution was further examined by Sanger sequencing of the remaining six evolved populations (R1–R6). The results showed that the mutations were highly concentrated after the first (R1) and fifth (R5) rounds of starvation, and most of the mutations were frameshift mutations that completely destroyed the gene products (Table 1). Among these ten mutations, three corresponded to translation functions (indicated by +), and the rest concerned genes for membrane and transport. None of these mutations or genes were reported in previous studies of *Escherichia coli* cells in stasis or slow growth, in which mutations are often observed in stress response regulators [24, 27, 35–37]. The significant accumulation of mutations in the membrane was consistent with the previous finding of the changes in porin expression under nutritional limitations [5, 38]. These transport-related mutations most likely caused the changes in membrane, perhaps, along with the survival trade-offs, as previously reported [28, 29, 39].

**Table 1:** Genome mutations accumulated from R1 to R7. The mutation accumulation is represented in color from R0 to R7, which indicate the rounds of repeated starvation and re-growth. 1 and 0 indicate the mutant and the wild type, respectively. Genome position, Change in DNA and Gene refer to AP012306 (DDBJ). Change in protein, Description and Cell location are according to GenoBase and RegulonDB. The mutations that occurred in the non-coding regions are indicated with parentheses in the “Cell location” column. The two mutations fixed in R1 were maintained in R2–R7. The additional mutations were detected in R5, R6, and R7. The cell location of the mutations indicated as outer/integral membrane or periplasmic suggest these mutations play a role in transport, and the mutations corresponding to *rnr*, *rpsI* and *rpsA* participate in translation. Asterisks and plus marks in Description indicate essential genes and gene functions related to translation.

Mutation accumulation								Genome position	Change in DNA	Change in protein	Gene	Description	Cell location
R0	R1	R2	R3	R4	R5	R6	R7						
0	1	1	1	1	1	1	1	2845628	G → T			+terminator region of rpsI* (ribosomal protein S9)	(Cytoplasmic)
0	1	1	1	1	1	1	1	2852633	GGCG → -	GlyGlu → frameshift	*argR*	DNA-binding transcriptional dual regulator	Cytoplasmic
0	0	0	0	0	1	1	1	359154	C → T	Trp → stop	*cyoA*	cytochrome o ubiquinol oxidase subunit II	Integral membrane
0	0	0	0	0	1	1	1	849900	A → G			activator and repressor region of *ompF*	(Outer membrane)
0	0	0	0	0	1	1	1	1112111	C → T	Gly → Glu	*cls*	cardiolipin synthase 1	Integral membrane
0	0	0	0	0	1	1	1	3401538	G → T	Glu → stop	*rffD*	UDP-N-acetyl-D-mannosaminuronic acid dehydrogenase	Periplasmic
0	0	0	0	0	1	1	1	3836946, 3836949	CG → -, G → T	ArgGlu → frameshift	*rnr*	+exoribonuclease R, RNase R	Cytoplasmic
0	0	0	0	0	1	1	1	826231	- → TG	Glu → frameshift	rpsA*	+ribosomal protein S1	Cytoplasmic
0	0	0	0	0	0	1	1	1395675	G → -	Ala → frameshift	*ydgR*	predicted transporter	Integral membrane
0	0	0	0	0	0	0	1	1997216	G → A			hisJp promoter region of *hisJ*	(Periplasmic)

Intriguingly, the present evolution experiment identified the translation-related mutations, in particular, the previously reported lethal mutation in the essential ribosomal protein S1. Nevertheless, the cells were alive. Taking into account the results of genome mutation analysis, further examinations were performed to determine whether and how the growth fitness and the translation machinery of the evolved strains changed. The dynamics of mutation accumulation suggested that substantial physiological changes might have occurred in R1 and R5 (Table 1). Thus, the further analyses in detail were performed mainly on four cell populations: R0, the ancestor; R1 and R5, the populations with the most accumulated mutations; and R7, the final evolved phenotype.

### Gain in tolerance to starvation and loss of propagation speed in rich conditions

**Fig 2:**
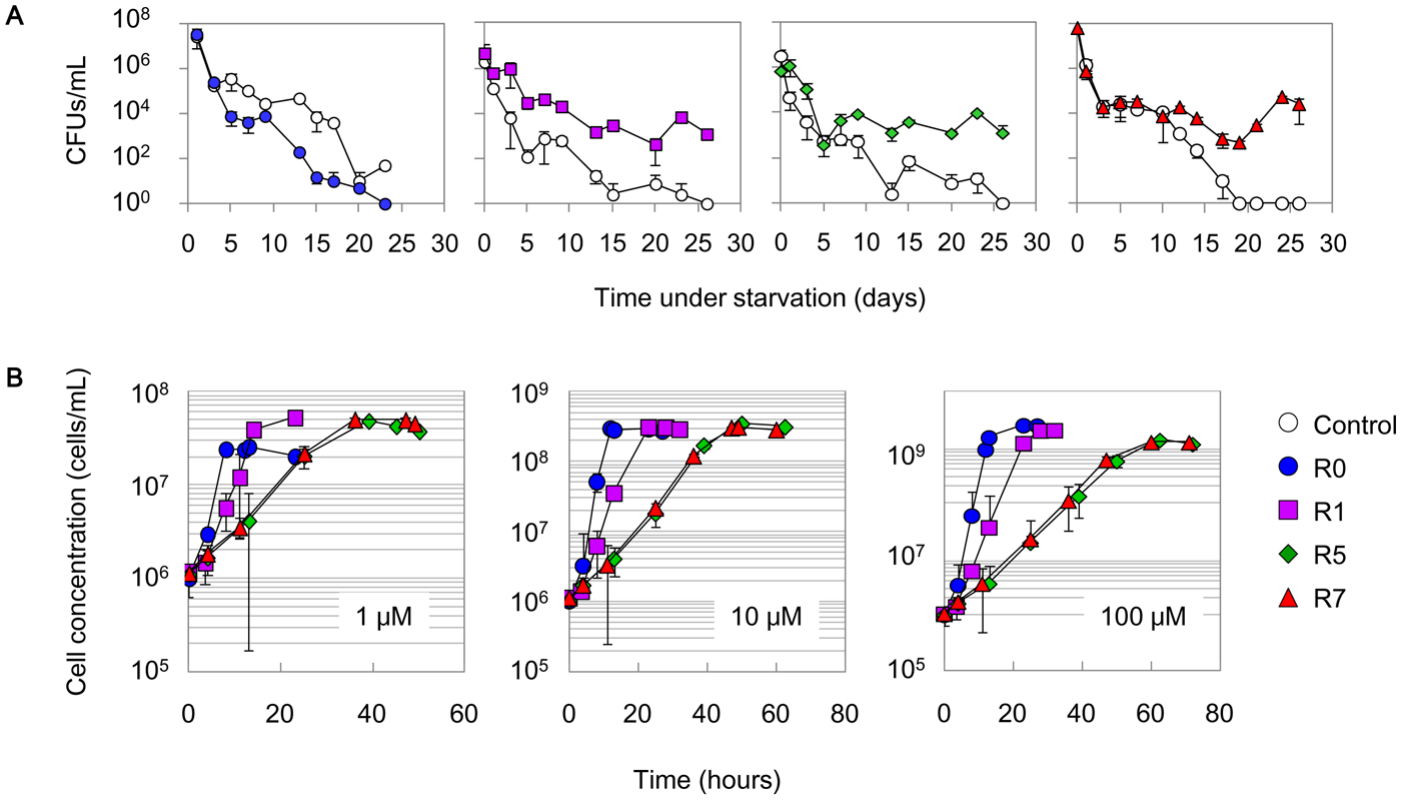
Reduced growth rate and improved sustainability. **A.** Competence under starved conditions. The control strain (open circles) was co-cultured with R0, R1, R5 and R7 under starved conditions. The temporal changes of cell concentrations were monitored by a CFU assay. The standard errors of four to eight assay plates are indicated. **B.** Growth in the presence of histidine. The temporal changes in cell concentrations of R0, R1, R5 and R7 in the presence of 1, 10, and 100 μM histidine were evaluated by flow cytometry. Blue circles, purple squares, green rhombuses and red triangles represent R0, R1, R5 and R7, respectively. The standard errors of three independent test tubes (cultures) are indicated.

Competition tests verified that the survivors exhibited stronger tolerances to starvation (Fig 2A, S2 Fig). The ancestor (R0) and the cells that experienced the starvation and resuscitation cycle (R1, R5 and R7) competed with the control strain, which was nearly equivalent to R0 except that it carried a different genetic marker (details in Materials and Methods). Both the control strain and R0 gradually lost their colony-formation ability throughout the competition (Fig 2A, open and blue circles). In comparison, followed by an initial CFU-decreasing phase, R1, R5 and R7 maintained relatively constant CFU values after approximately two weeks of starvation and finally outperformed the competition (Fig 2A; purple, green and red, respectively), consistent with reports on the cells that survived prolonged stationary phases [36, 40].

Unexpectedly, the precise growth dynamics tests showed that the evolved populations considerably reduced their propagation speed in histidine-rich conditions (Fig 2B). This decrease in growth fitness seemed to be gradual, as the starvation and re-growth cycle proceeded from R0 to R7 in the presence of 1–100 μM histidine (S3 Fig). According to a careful and repeated examination of the growth curves, we found that R0 had the best growth fitness, R1 grew more slowly, and R5 and R7 were equivalently slowest (Fig 2B), consistent with the timing of the mutation accumulation (Table 1). The results indicated that the improved sustainability under starvation came at the cost of growth deficiency under rich conditions.

### Improved capability of using trace amounts of resources for population increases

**Fig 3:**
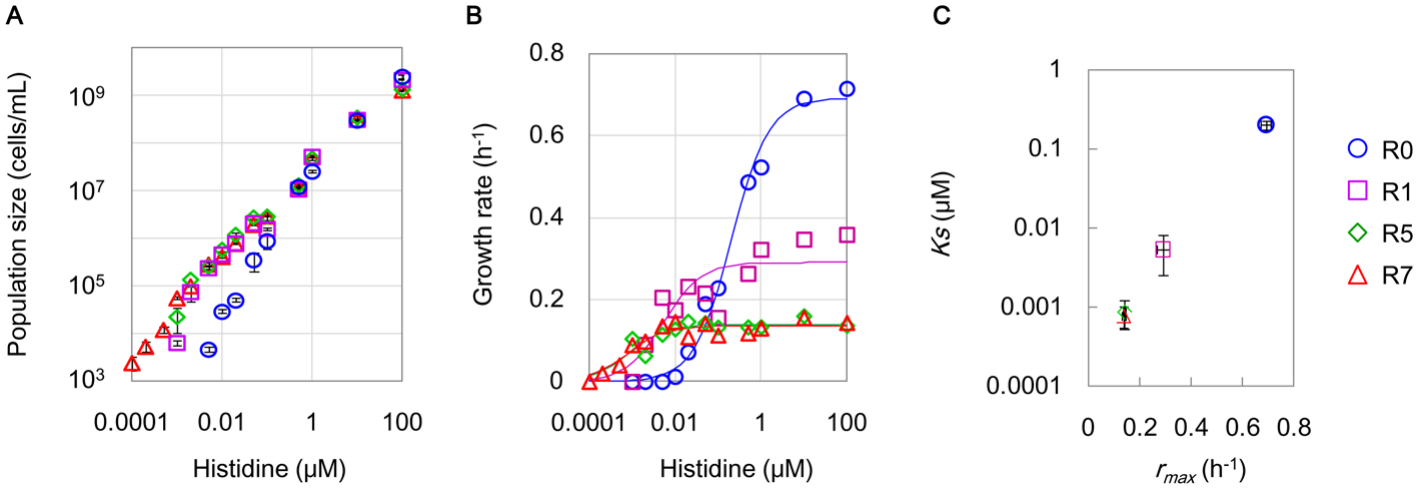
Growth rate and population size in response to various amounts of histidine. **A.** Saturated population densities detected by FCM. The maximal cell concentrations experimentally acquired in the various concentration of histidine are shown. Blue circles, purple squares, green rhombuses and red triangles represent R0, R1, R5 and R7, respectively. Standard errors of triplicates are indicated. **B.** Experimentally evaluated exponential growth rates. The growth rates of the growing cells were calculated according to the average cell concentrations of any three out of 12–15 test tubes at the defined time scales. The data fit to the Monod equation are shown as solid lines. Open marks represent the experimental measurements. The concentrations of histidine varied from 0.1 nM to 100 μM. **C.** Theoretically estimated *Ks* and *r*_*max*_. The parameters *Ks* (right) and *r*_*max*_ (left) were calculated according to the Monod equation using the experimentally acquired growth rates shown in B. The standard errors derived from the theoretical fitting are indicated.

Intensive measurements of cell growth in response to various amounts of histidine (0.1 nM–100 μM) showed that the cells became able to use trace amounts of histidine, and there was a positive correlation between the maximal population size and the supplemented histidine concentration (Fig 3A). R0 failed to increase the population size when the concentration of histidine was lower than 10 nM, whereas R1 and R7 maintained cell propagation even if the concentrations of histidine were as low as 2 and 0.2 nM, respectively (Fig 3A, S4 Fig). The lower limits of the histidine concentration required for a population increase were approximately 5- and 50-fold lower in R1 and R7, respectively, agreeing with the independent CFU assays (S4 Fig). In addition, the evolved saturated population sizes were remarkably amplified in nutrient-poor conditions (Fig 3A; <0.1 μM histidine). For instance, under feeding with 50 nM histidine, R1 and R7 reached population sizes larger than 10^6^ cells/mL compared with the population size of R0 of only ~10^5^ cells/mL (Fig 3A, S4 Fig), an increase of approximately one order of magnitude.

The evolved ability to utilize trace amounts of histidine for increasing the population coincided with a decrease in growth rates in rich conditions (Fig 3B). The growth rate of R0 was highly sensitive to the abundance of histidine (Fig 3B, blue circles), whereas the growth rates of R5 and R7 were nearly constant down to very low concentrations of histidine (Fig 3B; green rhombuses and red triangles, respectively). The trade-off in cell growth of a loss of growth in nutrient-rich (>0.1 μM) and a gain of growth in nutrient-poor (<50 nM) conditions indicated an evolutionary change of an increased cellular reproduction capability under resource limitations. A simple theoretical analysis illustrated the transition in the growth strategy for economical resource utilization. Fitting the experimentally evaluated growth rate data (Fig 3B, solid lines) with the Monod equation (see Materials and Methods) verified that the maximal growth rate (*r*_*max*_) decreased from ~0.69 to 0.14 h^-1^, and the substrate affinity (*K*_*S*_) was likely improved from ~0.2 to 0.0008 μM, under the conditions of repeated starvation and resuscitation (Fig 3C). Note that fitting the growth curves (Fig 2, S4 Fig) with the logistic equation, based on the *r/K* selection theory [41], drew an identical conclusion (S1 Note, S6 Fig). The cellular affinity for histidine transport or incorporation seemed to be enhanced, which might be attributed to mutations related to the membrane for transport. The decreased growth rate under nutrient-rich conditions occurred in exchange for the enhanced affinity for trace amounts of histidine for propagation under histidine-restricted conditions.

### Genetic disturbances in ribosomes for slowing translation

**Fig 4:**
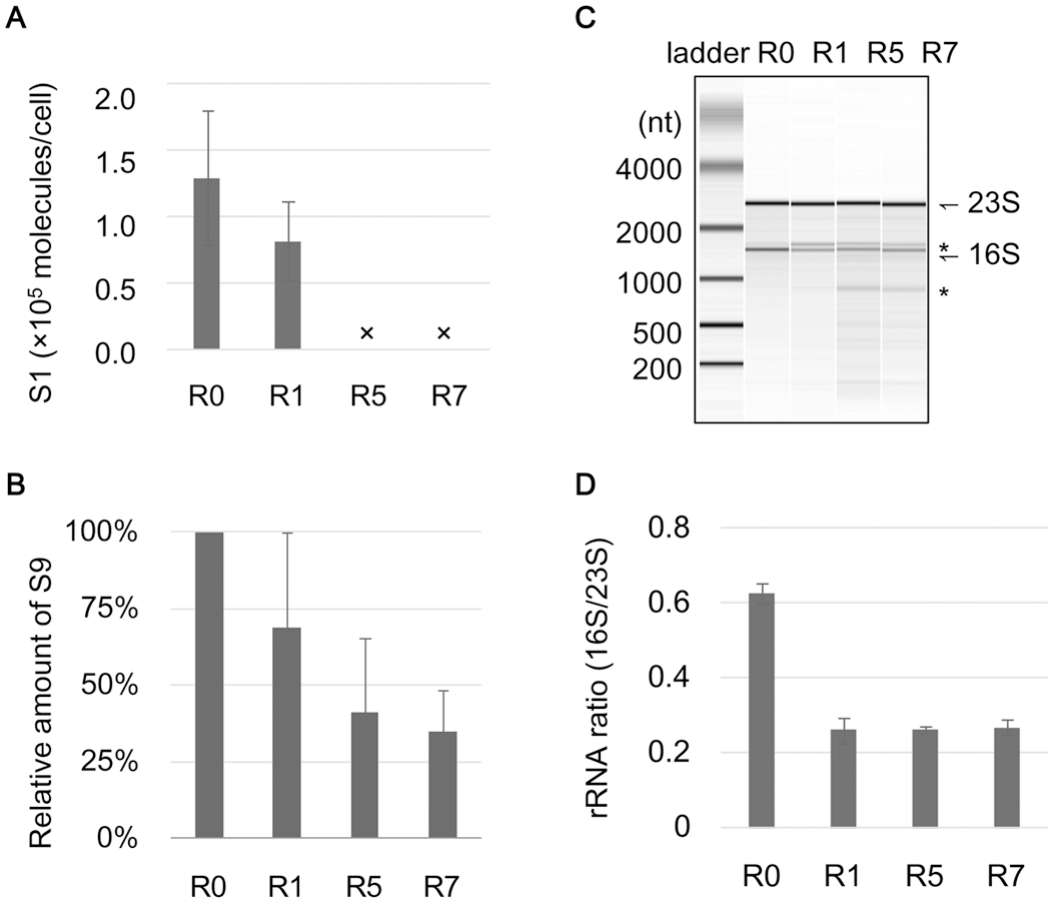
Damage in ribosomes. **A.** The relative abundance of the ribosomal protein S1 in cells. The ribosomal protein S1 was detected by western blotting, and its relative abundance was calculated according to the band intensity by image analysis using the purified S1 protein as the positive control. Crosses indicate the disappearance of the band. The standard errors of three independent Western blot analyses are shown. **B.** The relative abundance of the ribosomal protein S9. The ribosomal protein S9 was detected by western blotting, and its relative abundance was calculated according to the band intensity by image analysis. Standard errors of triplicates are shown. **C.** Ribosomal RNAs. An example RNA electrophoresis pattern of the total RNAs is shown. 16S and 23S rRNAs are indicated. The truncated rRNAs are indicated with asterisks. **D.** The ratio of 16S and 23S rRNAs. The ratios of 16S and 23S rRNAs were calculated according to the band intensity and area as shown in C. Standard errors of the RNA samples purified from three independent cell cultures are indicated.

Whether the reduced growth was attributed to genomic mutations in the ribosome was further investigated. The cell populations of R0, R1, R5 and R7 actively growing in the presence of histidine were collected (10^7^–10^8^ cells/mL) for protein and RNA analyses. Western blot analyses clearly demonstrated the loss of ribosomal protein S1 after R5 (Fig 4A, S5 Fig), indicating that the frameshift in the essential gene *rpsA* exclusively damaged the ribosomal protein S1, which associates with the 30S ribosome for initiating translation [42–44] and is known as one of 302 essential proteins among more than 4000 proteins in *E*. *coli* [45, 46]. The decreased cellular amount of the ribosomal protein S9 (Fig 4B) might coordinate the reduced growth rate [31, 47, 48]. This was probably due to a single nucleotide substitution on the terminator of *rpsI*, because the same substitution altered the transcription of S9 and S11 [49]. An analysis of ribosomal RNAs (rRNAs) identified truncated rRNAs (Fig 4C, asterisks) with irregular ratios of 16S/23S (Fig 4D) in the evolved slow-growing cells. The short rRNAs were more significant in R5 and R7, probably being attributed to the mutations in *rnr*, which participates in the quality control of structured RNAs [50, 51] and in ribosome degradation [52, 53]. These results suggested that both a decrease in ribosome abundance and a disorganization in the ribosome structural components had taken place in the evolved cells of active growth.

**Fig 5:**
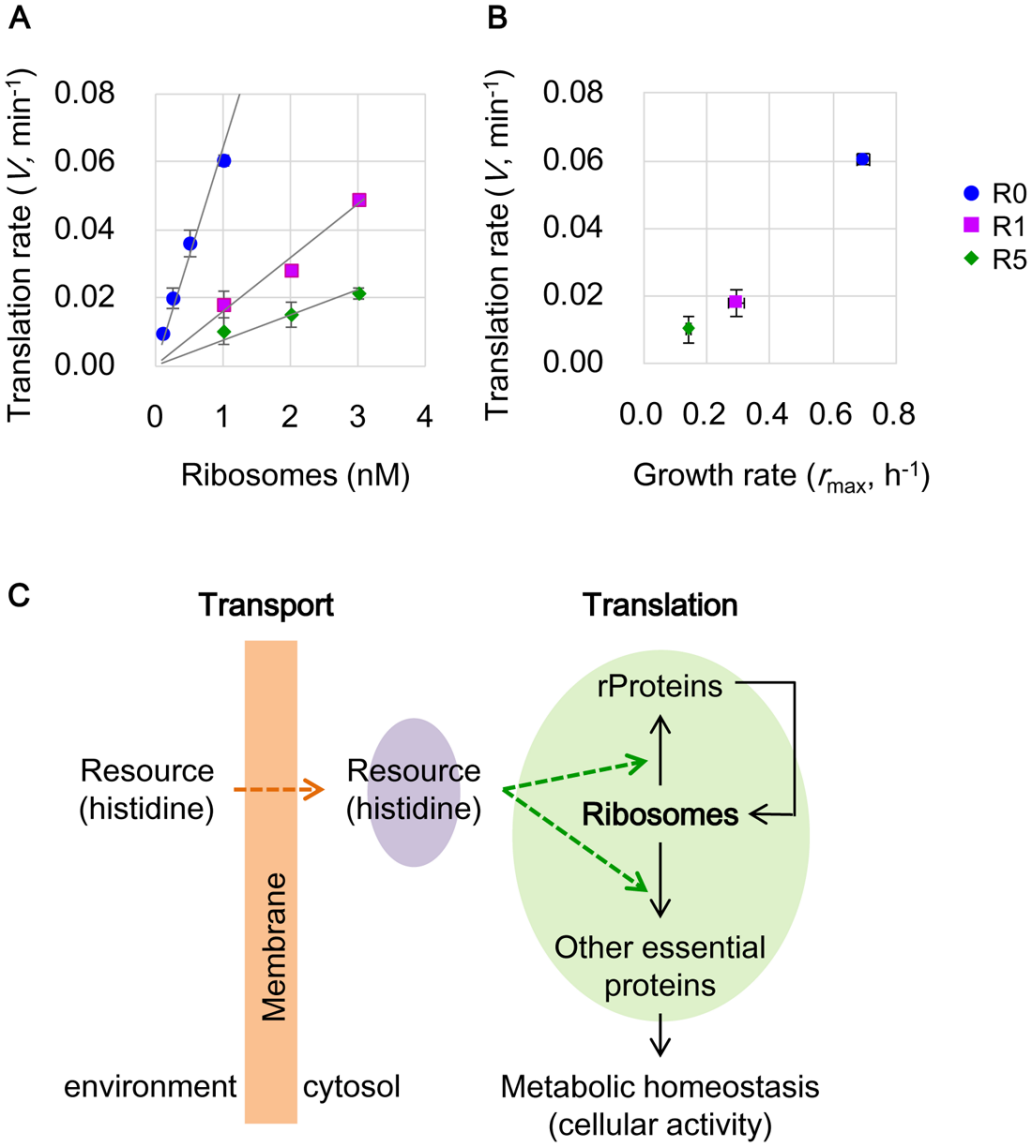
Relation between growth and translation. **A.** Translation activity of ribosomes. The maximal translation rates of the purified ribosomes were estimated according to GFP biosynthesis curves. The ribosomes from R0, R1 and R5 are indicated in blue, purple and green, respectively. Solid lines indicated linear regressions with the slopes of 0.064, 0.016 and 0.007 for R0, R1 and R5, respectively. The standard errors of repeated tests (n = 3–4) are indicated. **B.** Correlation between growth rate and translation rate. The translation rates at 1 nM ribosomes (data from A) are plotted against the theoretically estimated maximal growth rates *r*_*max*_ (data from Fig 3C). Blue, purple and red represent R0, R1 and R5, respectively. Standard errors are indicated as described in A and Fig 3C. **C.** A scheme of the changes in the growth economics for maintaining cellular activity. The purple shading represents the intracellular resource (histidine) concentration balanced between incorporation (orange arrow) and consumption (green arrows). The green arrows indicate the changes in resource (histidine) redistribution for translation under poor conditions. The green shading highlights the reorganized protein translation enabling survival. The orange shading suggests the changes caused by the mutations related to membrane and transport, which required further demonstration and were excluded from the present study.

*In vitro* translation analysis further demonstrated the correlation between the inactive translation of deficient ribosomes and the reduced growth rate. The translation activities of the purified R0, R1 and R5 ribosomes were evaluated by synthesizing a reporter protein (green fluorescent protein, GFP) in a component-reconstituted *in vitro* translation system (details in Materials and Methods). The translation activities of the R1 and R5 ribosomes were largely inhibited compared with those of R0 (Fig 5A). A linear regression of the ribosome-dependent translation rate (Fig 5A, solid lines) indicated that the ribosome activity was suppressed by almost one order of magnitude in R5, as the slope declined from ~0.06 (R0) to 0.007 (R5). The gradually decreased translation rates of the ribosomes (at a concentration of 1 nM) were positively correlated with the estimated maximal growth rates (Fig 5B). These results provided a direct link between the growth physiology and the activity of the translation machinery. The repeated starvation and resuscitation influenced the ribosomes to slow translation, resulting in the slow growth. The strategic change from fast to slow growth may prevent the resource pool from being exhausted, thereby preventing extinction from starvation (see Discussion). These results provide valuable information for the studies of slowly growing microbes in nature, similarly to the evolutionary studies that reported improved growth fitness [5–8].

## Discussion

Long-term experimental evolution occurring in recurrent favorable and hostile conditions, considering both growth and maintenance (Fig 1), resulted in an evolved nature of slow growth (Figs 2–3). The results demonstrated that it is possible to evolve for reduced growth fitness in addition to the fruitful demonstrations of evolving for increased growth fitness (reviewed in [2]). Slow growth, as a common tendency, was likely advantageous in adaptive evolution in a recurrent environment. Considering that slow growth was often reported in the cells that experienced stasis [27, 54] and could be naturally isolated [55], the strategy of running a slow life must be common for real microbial life in nature. Slow microbial life may successfully balance between growth and maintenance in response to irregular and unpredicted environmental changes. It is easy to understand that a high growth rate is able to enlarge the size of a population rapidly, improving a populations’ fitness. Alternatively, a resource-saving strategy could also increase or maintain the population size, which would be advantageous for habitat occupation and tolerance of the death of individuals.

One of the evolutionary consequences found in the present demonstration was the ultimate damage in the fitness component that was the main energy-consuming machinery (Figs 4–5). Translation repressed physiology, which is commonly stimulated by stringent responses or initiated at the stationary phase [56, 57] and is in coordination with amino acid starvation [58], appeared to be genetically fixed during the repeated starvation and resuscitation. Slow growth along with slow translational machinery has been observed in natural isolates of *E*. *coli* [55, 59], and the correlation between ribosomes and growth has been identified at both transcriptional and translational levels [31, 47, 48]. However, the adaptive evolution direct for a genetically inactive translation system was first reported here and was unexpected. It is unclear whether the starvation in histidine greatly induced the mutations related to translation because the long-term evolution of a transient stationary phase for a carbon source also resulted in mutations in translation [5].

Here, we proposed that the cellular maintenance under extreme resource limitations could benefit from slow translation. First, it was important to maintain a balance between incorporating histidine from environment (transport) and consuming histidine in protein (translation) in the cell. The total amount of the histidine resource was restricted in poor conditions, either outside or inside the cell. Highly active translation could rapidly use up the intracellular amino acids (*e*.*g*., histidine) and could cause an inclusive shutdown of translation and metabolic flux if the starvation conditions were not relaxed. Slowing translation facilitated equilibrating the intracellular histidine concentration (the charged tRNAs) and prevented resource exhaustion in the cells (Fig 5C, purple). Second, it was practical to redistribute the histidine resources in translation. Ribosome and protein synthesis accounts for more than 40% of the cellular total energy turnover [60], and the ribosomal proteins comprise 10–20% of the total cellular proteins [61]. Therefore, the damage to the ribosomes potentially allowed both resources (*e*.*g*., histidine) and energy, which were originally consumed for ribosome biosynthesis, to be used for the biosynthesis of other proteins (Fig 5C, green), similar to the allocation of resources for protein synthesis and growth [62]. Such a redistribution allowed the synthesis of the proteins required for maintaining a continuous metabolic flux [63]. Because even a dormant physiology maintains certain metabolic activities [9, 34], being alive required the translational and/or metabolic flux to occur in a continuous manner rather than in a pause-rebut manner. The population likely benefits from the slower production of proteins because it results in a more continuous, constant use of nutrient resources. Its byproduct appears to be the decreased total protein abundance per cell arising from redistributing the translation resources. This hypothesis was partially supported by the fact that the total protein amount decreased along with the regular translation of the reporter protein GFP in R1 and R7 (S7 Fig). The variety in the translational apparatus corresponding to diverse ecological strategies [64] may be the consequence of this property.

In addition, damaged ribosome-induced slow growth was accompanied by an increased affinity to histidine, indicating that the capacity of using trace amounts of resources played a role in survival under starvation. Additionally, studies on either natural isolates or laboratory strains have shown that bacteria can grow or adapt under trace amounts of a carbon source [18], and the change in membrane permeability played a role in a trade-off manner [39]. Accordingly, the transport- and membrane-related mutations detected in the present study might contribute to the membrane permeability for the improved capacity of transporting trace amounts of histidine from the environment (Fig 5C, orange). Although it is unclear whether this is a growth trade-off balanced between translation and transport, such improvements might allow the cells to use trace amounts of nutrients released from dead cells to prevent the extinction of the populations under extreme resource limitation. Whether and how the cells recycle the resources from dead cells needs to be further investigated, specifically with regard to the yield, death rate and turnover of the cells.

Note that the present study focused the changes eventually fixed in the final population. Because the mutation analysis was based on the final population R7, some mutations might have been missed in the intermediate populations (R1–R6). Considering the fact that the maximum population sizes were ~10^8^ cells/mL and the initial cell concentrations for resuscitation were approximately 10^2^–10^3^ cells/mL (Fig 1), no more than 20 generations occurred per round of re-growth. If a mutation only occurred during the growth step, the mutation rate would be approximately 10^−8^ per nucleotide per generation, which is as high as a mutator. The high mutation rate, in particular the burst in R5, may cause a heterogenetic population. Although heterogeneity is beyond the scope of the present study, we tested the colonies in the R7 population. Both reduced growth and a disappeared S1 were commonly detected in all single colonies (S8 Fig), indicating that the major genetic and physiological features within the population were highly equivalent or comparable. A number of studies reported heterogeneity in long-term starved cell populations [10, 25, 65] as an important strategy for adaptation [66]. We assumed that heterogeneity may burst during the hunger and/or starved phases, but the resuscitation (growth) phase favored the genotypes that had a proper physiology balanced between growth and maintenance.

In summary, this study presented a unique case of experimental evolution in recurrent starvation and re-growth conditions. An oscillating resource abundance triggered the cells to renovate their growth economics from time-saving to resource-saving, reflecting a balance between economy and effectiveness in biological homeostasis [67]. This evolutionary change was also assisted by the genetic repression of resource-consuming machinery, perhaps to minimize maintenance requirements [68] in severe environments. Although the mutations repeated observed in laboratory evolution sometimes failed to be detected in natural populations [69], the present experimental demonstration revealed the potential advantages of slow life for sustainability in severe nature and the slow translation might be one of the survival strategies.

## Materials and Methods

### Strains and cell culture

A previously constructed *E*. *coli* strain, *MDS42ΔhisF*::*Ptet-gfp-Km* [70], was used in the evolution experiments. The strain *MDS42*, holding a reduced genome, was originally constructed from the wild type *E*. *coli* MG1655 [71], and its genome sequence was previously analyzed and deposited (AP012306, DDBJ). As a control strain for the competition experiments, a strain carrying a similar genetic architecture, *MDS42ΔhisF*::*Ptet-rfp-Cm*, was newly constructed according to previous reports [70, 72]. Cells were cultured in 5 mL of minimal medium M63 (62 mM K_2_HPO_4_, 39 mM KH_2_PO_4_, 15 mM ammonium sulfate, 1.8 mM FeSO_4_7H_2_O, 15 mM thiamine hydrochloride, 0.2 mM MgSO_4_7H_2_O, and 22 mM glucose) supplemented with 100 μM histidine (Wako). The cells were incubated at 37°C in a bioshaker (BR-23FP, Taitec) with shaking at 200 rpm.

### Repeated starvation and resuscitation

Cells were initially grown in the presence of 10 μM histidine. After the cells reached saturation, the starvation step was initiated, leaving the cell culture in the same test tube. After starvation, re-growth was initiated by transferring a portion of the starved cells to the histidine-supplied medium (100-fold dilution). The remaining cell cultures were kept in stocks at -80°C (15% glycerol) for further analysis. The growth (re-growth) phase lasted two to seven days along with the cycles proceeding it. The cells were sampled during starvation for 1–2 months, mimicking the irregular periodic changes in nature. The periods of starvation were varied and were approximately 30 days in R1, R2 and R3; ~ 40 days in R4, R5 and R7; and ~ 60 days in R6. The elongated starvation periods in the later rounds were intended to enhance the selection pressure. Because the highly elongated period (~ 60 days) in R6 caused some difficulties in colony formation, the last round was returned to an intermediate starvation length of approximately 40 days. Time samplings of the starved cells for flow cytometry (FCM) analysis and colony formation unit (CFU) assays were performed at certain intervals, varying from one day to one month. A total of seven rounds of starvation and resuscitation cycles were performed (R1–R7). The cell cultures were shaken at 37°C throughout both starvation and resuscitation steps.

### CFU assay monitoring the experimental evolution

Cell cultures were sampled and, if necessary, diluted with fresh medium in various concentrations (by factors of 10 to 100,000) according to the presumed cell density. 100 μL of every culture or diluted sample was grown on three different types of agar plates. The plates were incubated at 37°C until the colonies were visible. A CFU assay, performed to monitor the process of the repeated starvation and re-growth, used three different plates: LB broth (Miller), LB supplemented with kanamycin (25 μg/mL), and M63 medium supplied with histidine (100 μM). The two types of LB plates were used as the indicators of contamination. The *E*. *coli* strain used in the present study could grow on an LB plate supplied with kanamycin because the strain carried a kanamycin resistant gene on its genome. If the CFU values counted from the two plates were incomparable, contamination may have occurred. The histidine-supplied M63 plate was used to monitor the population dynamics of the evolution process. Two to four replicates of these three agar plates were counted at each time sampling point.

### Competition experiments

The newly constructed *E*. *coli* strain, *MDS42ΔhisF*::*Ptet-rfp-Cm*, was used as the competitor. Four cell populations (R0, R1, R5 and R7) were used for the competition experiments. Cells were initially grown in the presence of 10 μM histidine separately. The nearly saturated cell cultures were subsequently mixed in equivalent volumes to a total of 10 mL, with initial concentrations of 10^7^–10^8^ cells/mL. The mixed cell cultures were further incubated in the same bioshaker at 37°C and subjected to time sampling at time intervals of one day to one week. The population dynamics under starvation, based on the CFU assay, were monitored for approximately one month. The agar plates for the CFU assay were prepared with M63 medium in the presence of 1 mM histidine. Multiple dilution rates with triplicates were used at each time sampling point. The colonies formed on the agar plates containing 25 μg/mL kanamycin (Wako) were identified as R0, R1, R5 and R7, and those formed on the agar plates containing 30 μg/mL of chloramphenicol (Wako) were assigned as the competitor strain. Two independent competition tests were performed. Approximately 3,000 plates were used for the competition experiments.

### FCM analysis

The cell concentrations and cellular GFP were evaluated with a flow cytometer (CantoII; Becton, Dickinson and Company) equipped with a 488-nm argon laser and a 515–545-nm emission filter (FITC). The following PMT voltage settings were applied: forward scatter (FSC), 400; side scatter (SSC), 400; and FITC, 600. The flow rates for the sample measurements were set from ‘low’ to ‘high’ according to the cell concentration. The cell concentrations were calculated according to the ratio of gated particles representing the number of *E*. *coli* cells carrying the reporter gene *gfp* and beads of known concentrations as previously described [70, 73]. Repeated measurements of independent cultures (n = 3–4) on different days were performed, and the averaged mean values are presented in the main text.

### Cell growth and maximal population sizes

The cell growth of R0–R7 in the presence of varied concentrations (0.1 nM–100 μM) of histidine was precisely evaluated, which was the most exhaustive experimental procedure in the present study. The precultures were all strictly controlled in the exponential growing phase according to the repeated tests for the preliminary prediction of growth. Precultures, gradually reducing the amount of histidine (from 1 μM to 1 nM), were repeatedly performed to examine the growth in the presence of low concentrations of histidine (<50 nM). Multiple cultures (12–16 test tubes) were applied for monitoring the growth curves under each condition. Temporal samplings of the cell cultures were performed, and the cell concentrations were evaluated by flow cytometry as described in FCM analysis. To record the nearly maximal population sizes properly, we used the highest cell concentrations after the cell cultures transitioned from the exponential to the stationary phase. The growth rates were calculated according to the following formula: ln(Ct/C0)/t, where Ct, C0 and t represent the final and initial cell concentrations (cells/mL) within the exponential phase and the culture time (hours), respectively.

### Theoretical analysis

The experimentally determined growth rates of R0, R1, R5 and R7 were fit to the Monod equation (Eq 1) using the customized script in R [74] to determine the relation between the growth rate and the initial concentration of histidine.

$$r=r_{\text{max}}\frac{S}{K_{S}+S}$$ (1)

where *S*, *r*, *r*_*max*_ and *K*_*S*_ represent the substrate concentration (histidine, μM), growth rate (h^-1^), maximal growth rate (h^-1^) and substrate concentration at which the growth rate was half of r_max_ (μM), respectively. The mean values of the growth data acquired by triplicate tests were used in the theoretical analysis and in the estimated values of all parameters. A theoretical analysis using both the logistic and Monod equations, based on *r/K* selection, can be found in S1 Note.

### DNA sequencing and mutation determination

The genomes of the ancestor R0 and the evolved cell population R7 were resequenced using a next-generation sequencer (Junior, Roche). Genomic DNA was purified with a Wizard Genomic DNA Purification kit (Promega) and fragmented using a DNA shearing system (Covaris), according to the manufacturer’s instructions. Whole-genome shotgun sequencing by the 454 GS Junior platform (Roche) was performed according to the manufacturer’s instructions. The sequence reads were assembled using Newbler 2.7 and aligned using the GS Reference Mapper software (ver. 2.6; Roche). Approximately 99% of the reads in each dataset (DRA003743, DDBJ) were uniquely mapped to the MDS42 genome (AP012306, DDBJ). The mutations were analyzed using the GS Reference Mapper software. The candidate mutations were further determined by Sanger methods of the genome samples subjected to a resequencing analysis using a genetic analyzer (ABI PRISM 3100, Applied Biosystems). Sequencing was performed to both the purified genomes and the cell pellets of these populations. In addition, the cell populations (R0–R7) acquired in the experiments of growth curves were repeatedly sequenced for further verification.

### RNA purification and analysis

*E*. *coli* cells exponentially growing in the presence of 1, 10 and 100 μM histidine were collected (10^7^–10^8^ cells/mL) for repeated tests. The total RNA was purified using a commercial kit (RNeasy, Qiagen), as described in detail elsewhere [73]. The purified RNA was analyzed with an RNA analysis kit (RNA 6000 nano chip, Agilent Technology) for sizing and quantification (2100 Bioanalyzer, Agilent Technology) according to the manufacturer’s instructions.

### Proteins and antibodies

The purified ribosomal protein S1 and its monoclonal antibody (mouse IgG) were previously prepared [75]. A histidine-tagged green fluorescent protein (GFP-His) was purified using the same protocol [76] for purifying the enzymes present in the component-reconstituted cell-free translation system [77]. The polyclonal antibody to GFP (rabbit IgG) was purchased from Funakoshi. The peptide (97–114 amino acids, based on peptide prediction) of the ribosomal protein S9 was chemically synthesized (Sigma), and its antibody (rabbit serum) was produced commercially (Sigma). The anti-mouse and anti-rabbit IgG HRP conjugates were purchased from R&D Systems.

### Protein assay and western blotting

Cells were collected by centrifugation at 10,000 g for 2 min and were suspended with BugBuster protein extraction reagent (Novagen) for protein extraction according to the manufacturer's instructions. The supernatant of the cell crude extract was subjected to a protein assay (Pierce BCA protein assay reagent, Thermo Fisher Scientific) according to the manufacturer’s protocol. SDS-polyacrylamide gel electrophoresis (SDS-PAGE) of cell pellets, which were suspended with phosphate-buffered saline (PBS) buffer (Sigma), was performed with a precast gel (12% Mini-PROTEAN TGX Precast Gel, Bio-Rad) and was blotted with a semidry system (Trans-Blot Turbo Transfer System and mini PVDF Transfer Pack, Bio-Rad). The proteins were detected with primary antibodies (0.1–0.5 μg/mL) derived from mice or rabbits followed by the relevant IgG-HRP conjugates (1,000- to 5,000-fold dilution). After development with a chemiluminescent substrate (Advance Western Blotting Detection Kit, GE Healthcare), the proteins were detected using a luminescent imaging analyzer (Image Quant 350, GE Healthcare). The standard curves for the quantitative evaluation of cellular GFP and S1 ranged from 1–50 ng per lane. The number of cells loaded per lane varied from 1×10^6^ to 2×10^7^ cells according to the preliminary estimated sensitivity in detection.

### Ribosome purification

*E*. *coli* 70S ribosomes were purified according to a previous report [78] with the following modifications. The cells were always grown in the M63 minimal medium supplied with 100 μM of histidine. The cell stocks (R0, R1 and R5) were initially inoculated in 5 mL of media and cultured for 16–88 h, as the growth rates of the cells largely varied. Subsequently, the 5 mL initial cultures were all transferred to 250 mL of fresh media and cultured for 8–24 h as precultures. Finally, these precultures were transferred to 2–4 L of fresh media and cultured until the absorbance at OD600 reached 0.5–0.6. The cells were collected by centrifugation (CR21G, HITACHI) at 15,000 g for 10 min, and cell extraction was performed using a Multi-Beads Shocker (Yasui Kikai). In the chromatography step (HiTrap Butyl FF, GE Healthcare), the fractions of high absorbance (OD260 >1.5) were collected as the ribosomes.

### *In vitro* translation

Cell-free translation was performed using a component-reconstituted system, named the PURE system [77], in which the elements participating in the translation reaction could be freely adjusted. The enzymes constituting the PURE system were purified as previously described [76]. All the components comprising the PURE system were highly modified for improved translation productivity and were prepared as previously reported [79]. This highly tuned component-reconstituted *in vitro* translation system (PURE system), comprising all 20 amino acids (0.2 mM), was used in the presence of the purified ribosomes R0, R1 or R5. The translation activity was evaluated with a reporter gene, *gfp* (green fluorescent protein), as previously reported [79, 80]. The mRNAs of *gfp* were transcribed in vitro and purified for translation in the PURE system. The translation reactions were performed in 20 μL volumes in 96-well plates (MicroAmp Optical, Applied Biosystems) and repeated in the presence of varied concentrations of ribosomes (0.1–3 μM). The translation reactions were monitored with a real-time PCR system (Mx3005P, Agilent Technology), and the data were normalized by an intrinsic fluorescent maker as previously reported [79].

## Supporting Information

S1 Fig Three lineages of repeated starvation and resuscitation. The panels from top to bottom, indicated with blue-, orange-, brown- and black-filled circles, stand for the FCM count, the CFU assay on the agar plates of the histidine supplied M63, the kanamycin-containing LB and the LB, respectively. The dilution points were omitted. The L1 lineage (as shown in Fig 1B) was used for further analysis in the main text. The dilution points were omitted.
(TIF)

S2 Fig Competence under starved conditions. The repeated tests as same performed in Fig 2A. The control strain (open circles) was co-cultured with R0, R1, R5 and R7 under starved conditions. The temporal changes of cell concentrations were monitored by a CFU assay. Standard errors of four to eight assay plates are indicated. Blue circles, purple squares, green rhombuses and red triangles represent R0, R1, R5 and R7, respectively. Standard errors of four to eight assay plates are indicated.
(TIF)

S3 Fig Growth curves in the presence of histidine. Temporal changes in the cell concentrations of R0, R1 R2, R4, R5, R6 and R7 in the presence of 1, 10, and 100 μM of histidine are shown in varying colors. The data sets of R0, R1, R5 and R7 were used for Fig 2B. Standard errors of three independent test tubes are indicated.
(TIF)

S4 Fig Cell growth in limited nutrition. **A.** Growth curves with trace amounts of histidine. Temporal changes in the cell concentrations of R0, R1 and R7 in the presence of trace amount of histidine varying from 0 to 50 nM. The cell concentrations were evaluated by flow cytometry. Standard errors of triplicates are indicated. **B.** The lower limit of the histidine concentration for cell propagation. Cell concentrations of R0, R1 and R7 grown in trace amount of histidine (0–10 nM) were re-estimated by a CFU assay. Samplings at three time points were performed as shown in the order of black, gray and white bars. The three time points for R0, R1 and R7 were 0, 10 and 22 h; 0, 24 and 46 h; and 0, 22.5 and 46.5 h, respectively. Standard errors of three to four assay plates are indicated.
(TIF)

S5 Fig Western blotting of S1. **A.** Example image example of western blotting detecting S1 in R0–R7. **B.** Cellular protein abundance of S1. The cellular concentrations of S1 are calculated according to repeated tests, and the results of R0, R1, R5 and R7 are used in Fig 4A. The standard errors of repeated tests (n = 3–4) are indicated.
(TIF)

S6 Fig Precise estimation of *Ks* and *r*_*max*_. **A.** The relationship between *r* and *K*. The parameters of *r* and *K* that were estimated by fitting the growth data sets with the logistic equation are shown. Only the well-fitted results are shown, and those without growth are indicated as zero. **B.** The relationship between the histidine concentration and the estimated growth rate. The estimated growth rates are from A. Data fitted with the Monod equation are shown in the solid lines. **C.** Estimated *Ks* and *r*_*max*_. The parameters of *r*_*max*_ and *K*_*S*_ that were estimated by data fitting with the Monod equation are shown. **D.** Yield *vs* growth. The ratios between *r*_*max*_ and *K*_*S*_ are calculated. Blue circles, purple squares and red triangles indicate R0, R1 and R7, respectively. The standard errors of the theoretical estimations are indicated.
(TIF)

S7 Fig Protein biosynthesis in R0, R1 and R7. **A.** Total protein abundance. The total amounts of soluble proteins were evaluated by a BCA assay. **B.** GFP translation. Western blotting of the cellular GFP was performed as described in S7 Fig. The *gfp* reporter was chromosomally incorporated and regulated by a constitutively expressed promoter P_tet_. The standard errors of repeated tests (n = 3–4) are indicated.
(TIF)

S8 Fig Characterization of R7 colonies. **A.** Growth of R7 colonies. The initial cell concentrations were determined after the inoculation of the colonies in the liquid media 4.5 h later, and the cell concentration was time-sampled by flow cytometry. The selected 16 colonies were numbered from C1 to C16. The colors represent the size of colonies roughly estimated by eye. Red, green, orange and purple represent 16 successful colonies of large, medium, small and tiny sizes, respectively. **B.** Western blot of ribosomal protein S1. Western blotting was performed to detect S1 in 16 single colonies, corresponding to the colonies in A. The results showed that the S1 protein had disappeared in all 16 colonies, supporting the results of identical growth rates. The arrow indicates the S1 position. c1-c16, wt, and 70S represent the 16 single colonies, the wild type *E*. *coli* strain, and the purified wild type ribosome 70S, respectively. R0 and R7 indicate the two cell populations.
(TIF)

S1 Note Supplementary note I is supplied in a single PDF file. (PDF)
