# Supplementary figures and images for: Evolutionary Consequence of a Trade-Off between Growth and Maintenance along with Ribosomal Damages
Source: PLoS One. 2015 Aug 20;10(8):e0135639. doi: 10.1371/journal.pone.0135639 (PMC4546238; doi:10.1371/journal.pone.0135639)

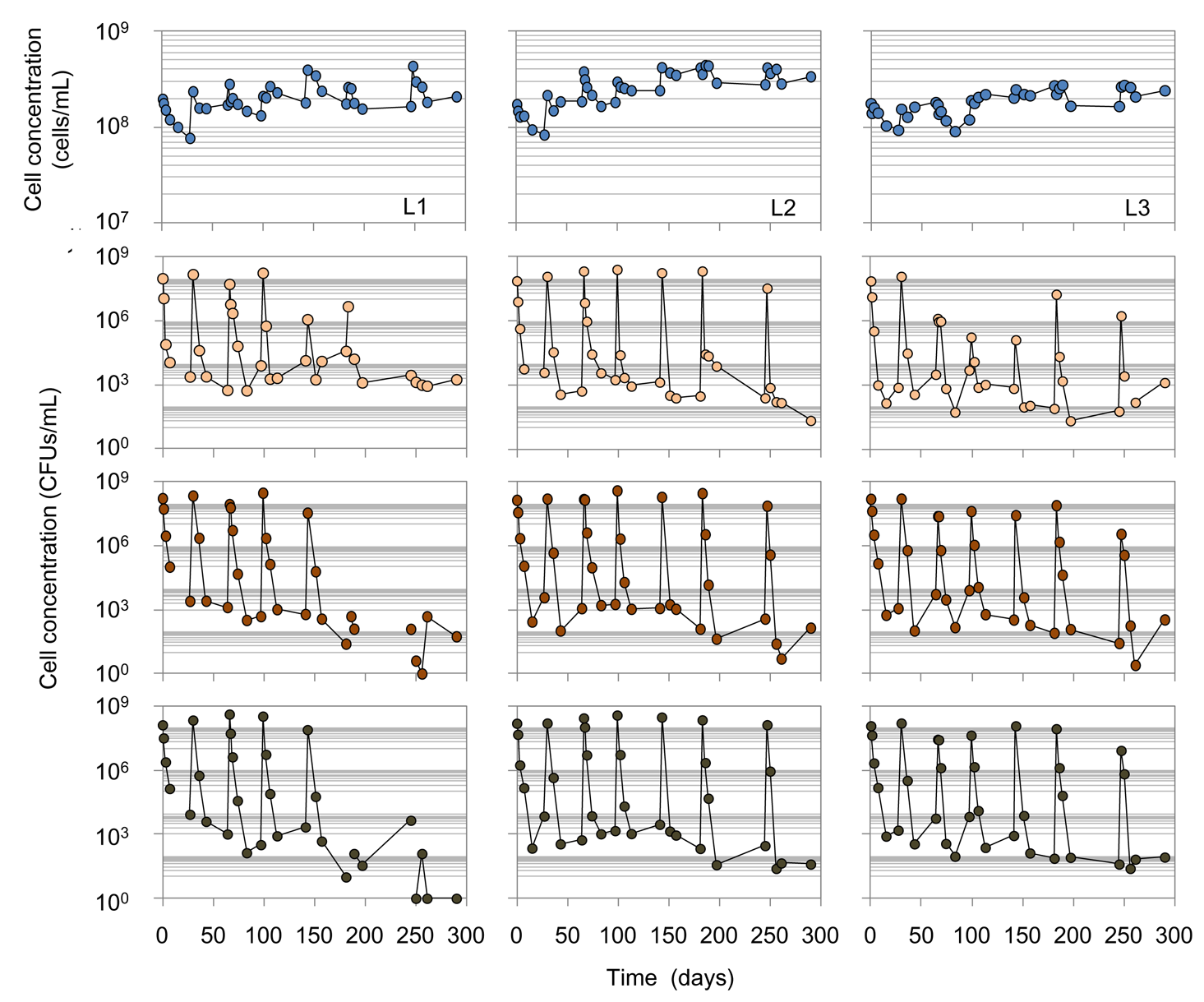

Supplement: S1 Fig — The panels from top to bottom, indicated with blue-, orange-, brown- and black-filled circles, stand for the FCM count, the CFU assay on the agar plates of the histidine supplied M63, the kanamycin-containing LB and the LB, respectively. The dilution points were omitted. The L1 lineage (as shown in Fig 1B) was used for further analysis in the main text. The dilution points were omitted. (TIF) [file pone.0135639.s001.tif]

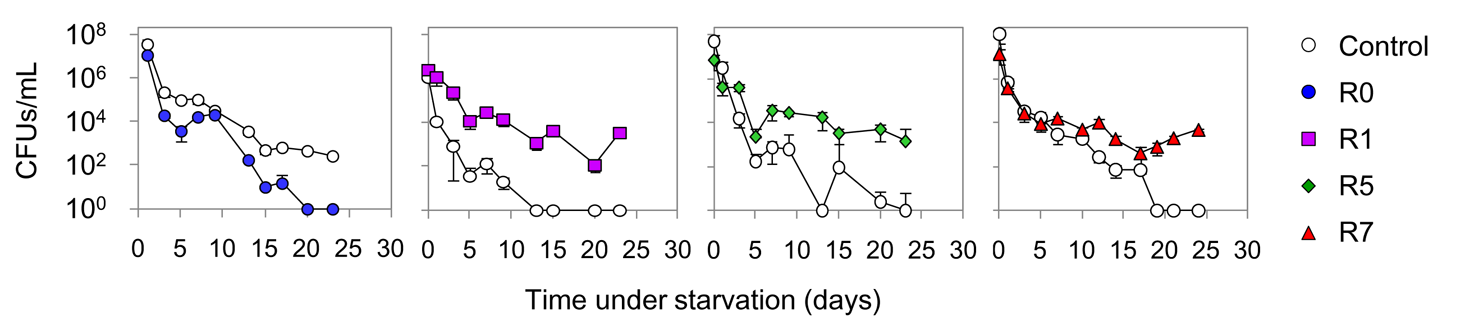

Supplement: S2 Fig — The repeated tests as same performed in Fig 2A. The control strain (open circles) was co-cultured with R0, R1, R5 and R7 under starved conditions. The temporal changes of cell concentrations were monitored by a CFU assay. Standard errors of four to eight assay plates are indicated. Blue circles, purple squares, green rhombuses and red triangles represent R0, R1, R5 and R7, respectively. Standard errors of four to eight assay plates are indicated. (TIF) [file pone.0135639.s002.tif]

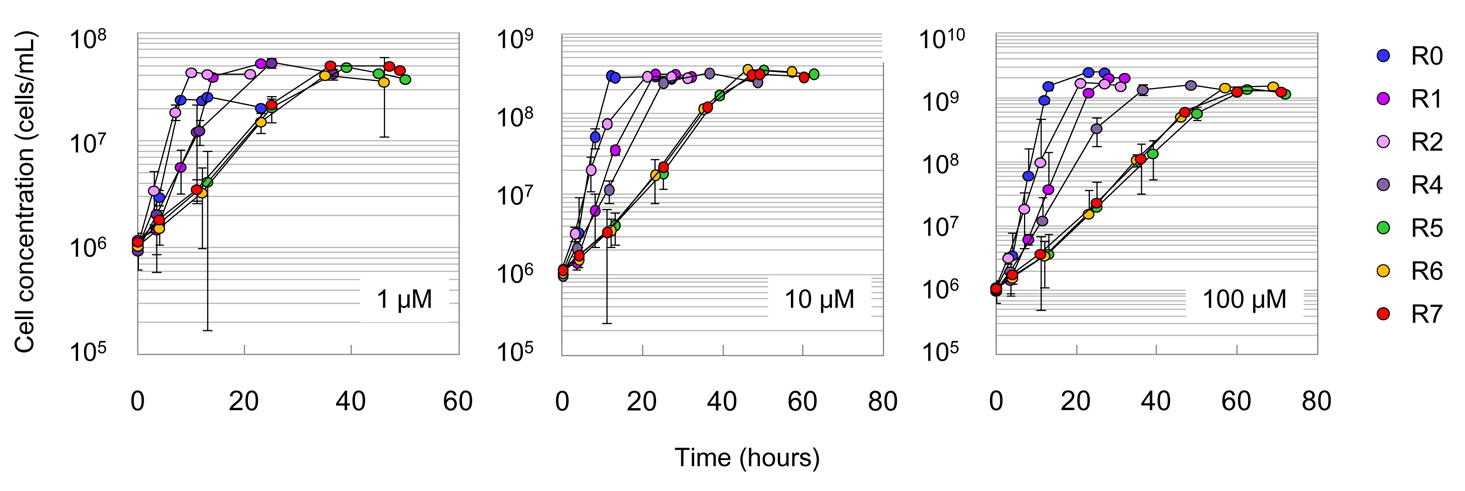

Supplement: S3 Fig — Temporal changes in the cell concentrations of R0, R1 R2, R4, R5, R6 and R7 in the presence of 1, 10, and 100 μM of histidine are shown in varying colors. The data sets of R0, R1, R5 and R7 were used for Fig 2B. Standard errors of three independent test tubes are indicated. (TIF) [file pone.0135639.s003.tif]

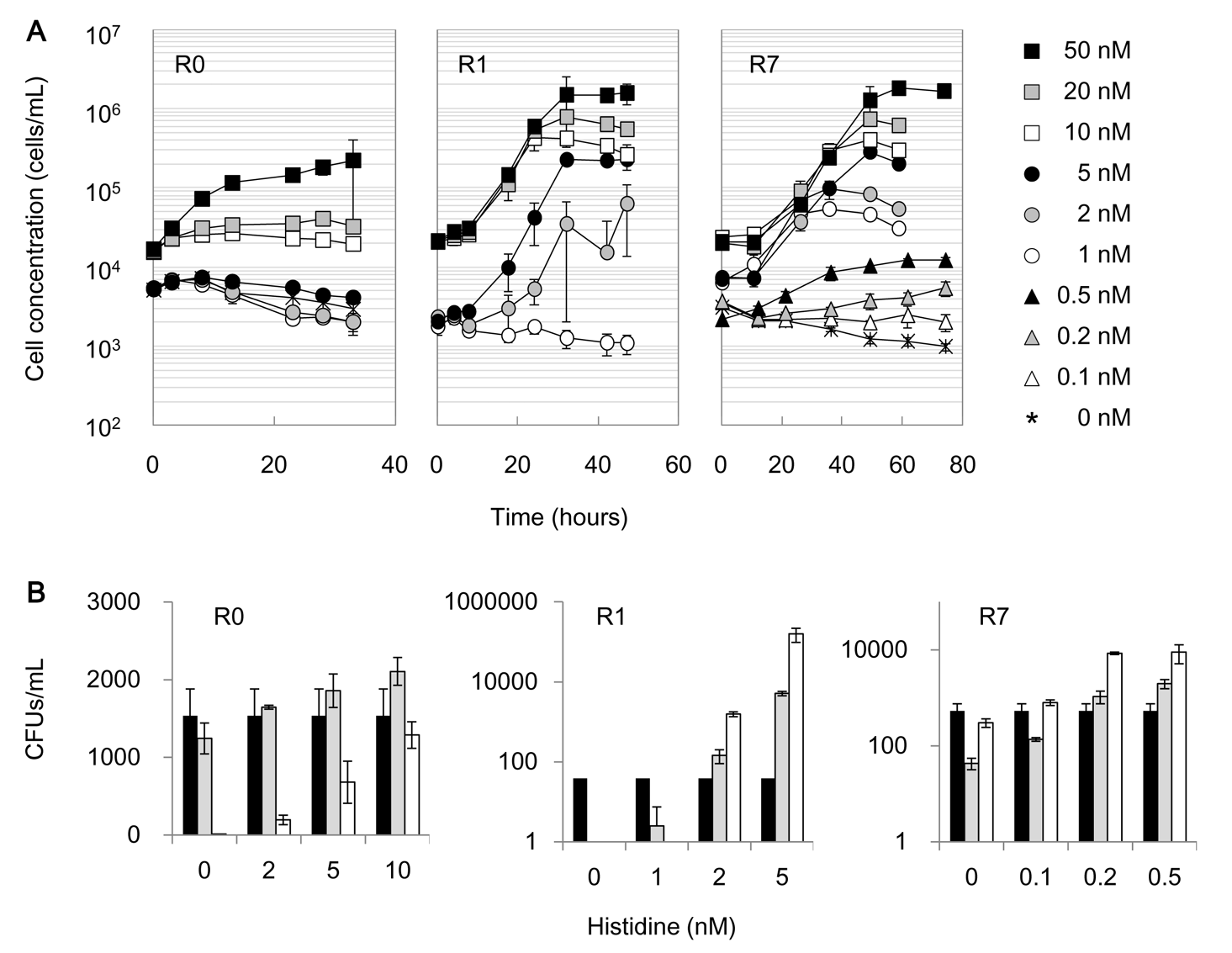

Supplement: S4 Fig — A. Growth curves with trace amounts of histidine. Temporal changes in the cell concentrations of R0, R1 and R7 in the presence of trace amount of histidine varying from 0 to 50 nM. The cell concentrations were evaluated by flow cytometry. Standard errors of triplicates are indicated. B. The lower limit of the histidine concentration for cell propagation. Cell concentrations of R0, R1 and R7 grown in trace amount of histidine (0–10 nM) were re-estimated by a CFU assay. Samplings at three time points were performed as shown in the order of black, gray and white bars. The three time points for R0, R1 and R7 were 0, 10 and 22 h; 0, 24 and 46 h; and 0, 22.5 and 46.5 h, respectively. Standard errors of three to four assay plates are indicated. (TIF) [file pone.0135639.s004.tif]

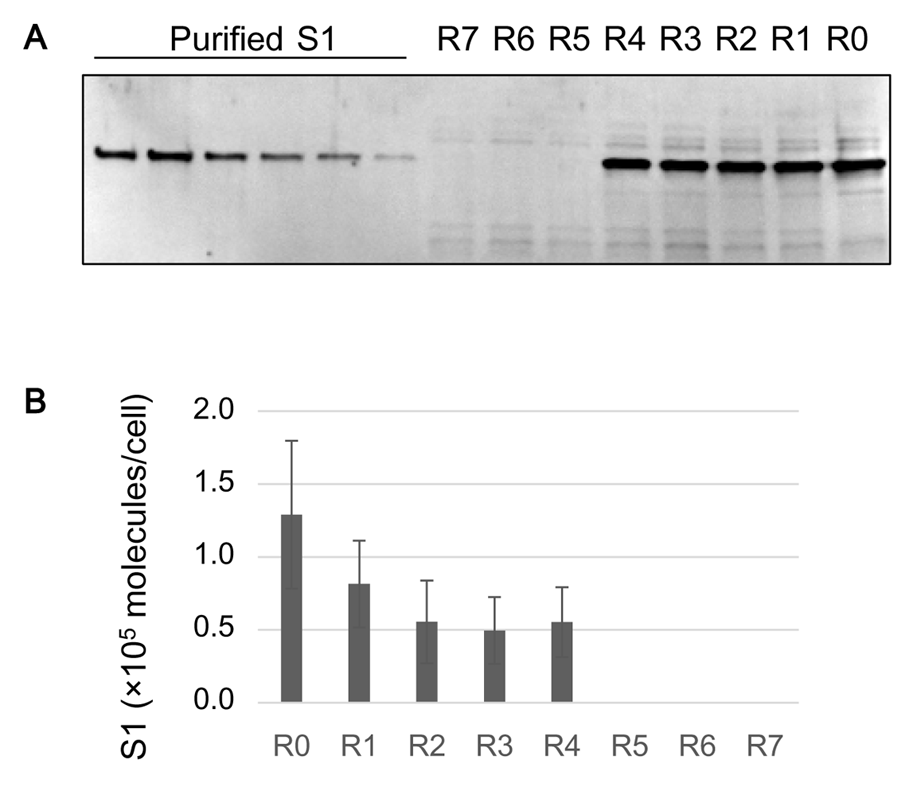

Supplement: S5 Fig — A. Example image example of western blotting detecting S1 in R0–R7. B. Cellular protein abundance of S1. The cellular concentrations of S1 are calculated according to repeated tests, and the results of R0, R1, R5 and R7 are used in Fig 4A. The standard errors of repeated tests (n = 3–4) are indicated. (TIF) [file pone.0135639.s005.tif]

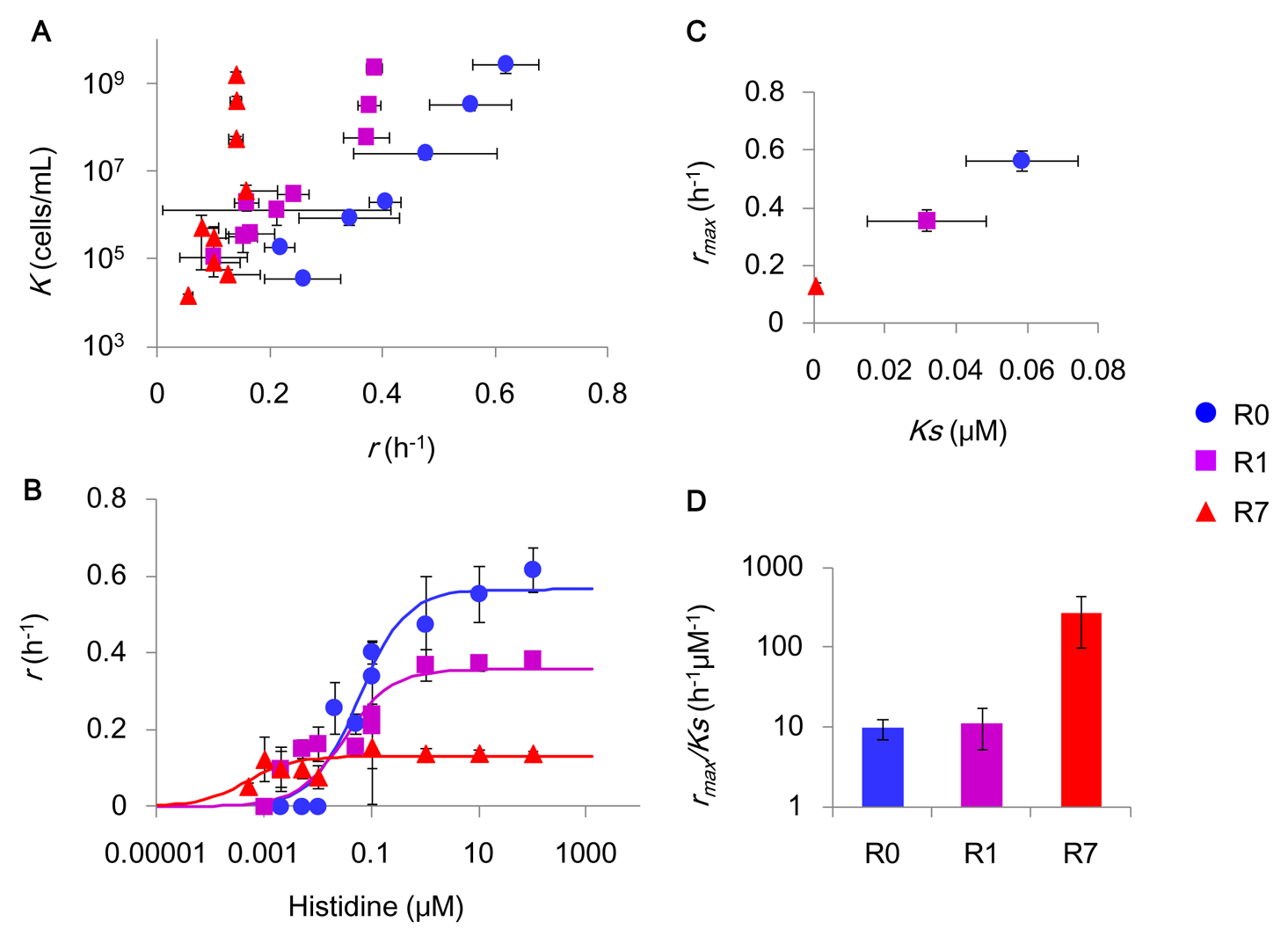

Supplement: S6 Fig — A. The relationship between r and K. The parameters of r and K that were estimated by fitting the growth data sets with the logistic equation are shown. Only the well-fitted results are shown, and those without growth are indicated as zero. B. The relationship between the histidine concentration and the estimated growth rate. The estimated growth rates are from A. Data fitted with the Monod equation are shown in the solid lines. C. Estimated Ks and r max. The parameters of r max and K S that were estimated by data fitting with the Monod equation are shown. D. Yield vs growth. The ratios between r max and K S are calculated. Blue circles, purple squares and red triangles indicate R0, R1 and R7, respectively. The standard errors of the theoretical estimations are indicated. (TIF) [file pone.0135639.s006.tif]

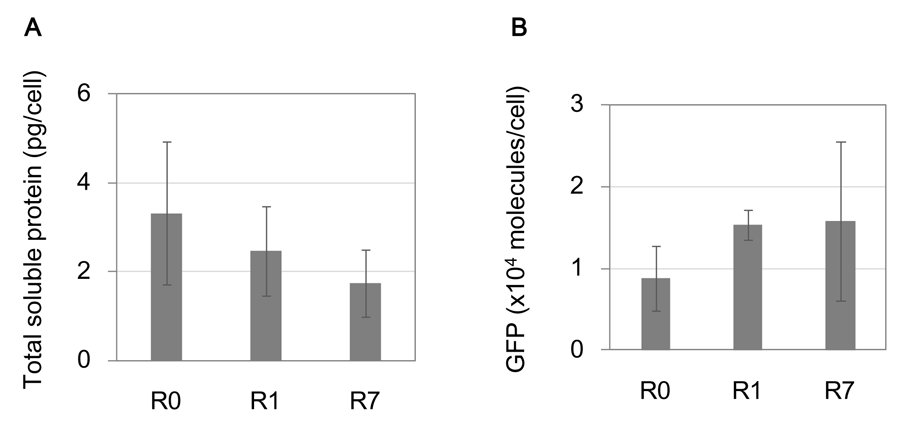

Supplement: S7 Fig — A. Total protein abundance. The total amounts of soluble proteins were evaluated by a BCA assay. B. GFP translation. Western blotting of the cellular GFP was performed as described in S7 Fig. The gfp reporter was chromosomally incorporated and regulated by a constitutively expressed promoter Ptet. The standard errors of repeated tests (n = 3–4) are indicated. (TIF) [file pone.0135639.s007.tif]

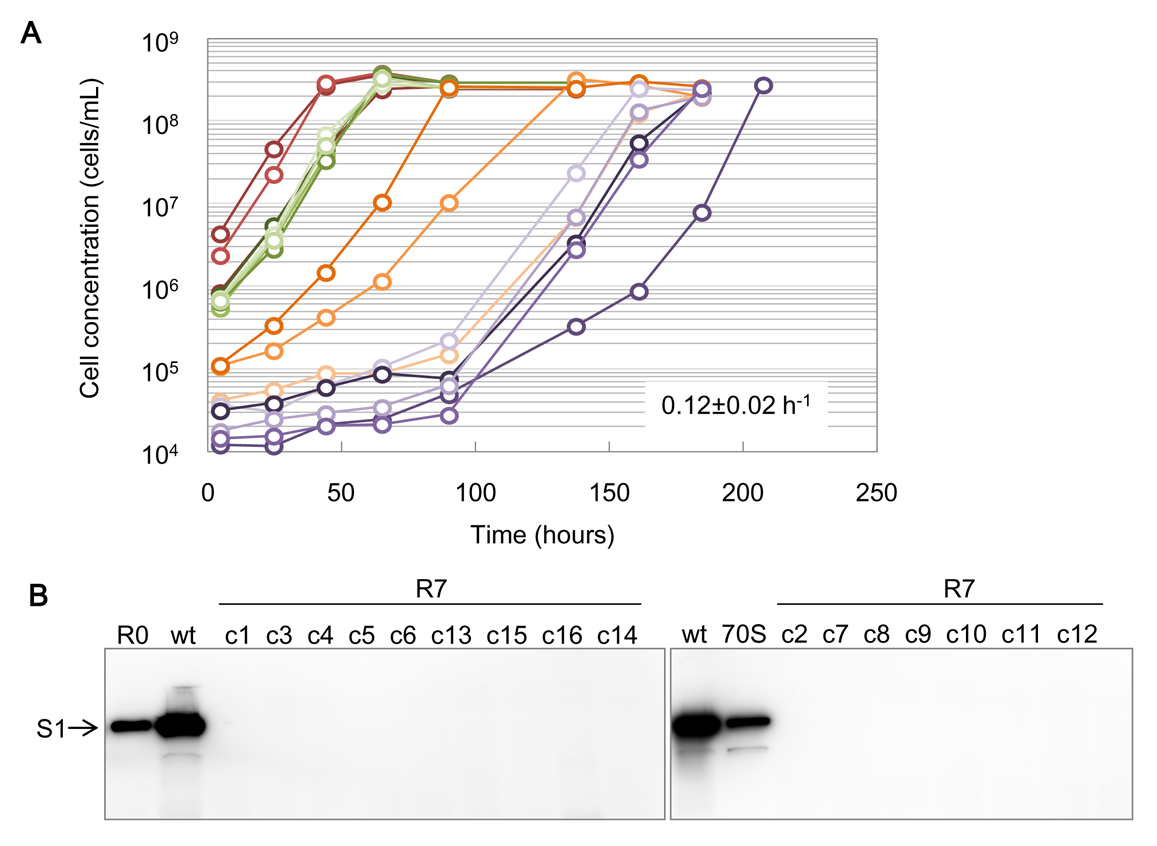

Supplement: S8 Fig — A. Growth of R7 colonies. The initial cell concentrations were determined after the inoculation of the colonies in the liquid media 4.5 h later, and the cell concentration was time-sampled by flow cytometry. The selected 16 colonies were numbered from C1 to C16. The colors represent the size of colonies roughly estimated by eye. Red, green, orange and purple represent 16 successful colonies of large, medium, small and tiny sizes, respectively. B. Western blot of ribosomal protein S1. Western blotting was performed to detect S1 in 16 single colonies, corresponding to the colonies in A. The results showed that the S1 protein had disappeared in all 16 colonies, supporting the results of identical growth rates. The arrow indicates the S1 position. c1-c16, wt, and 70S represent the 16 single colonies, the wild type E. coli strain, and the purified wild type ribosome 70S, respectively. R0 and R7 indicate the two cell populations. (TIF) [file pone.0135639.s008.tif]
