# Supplementary material for: Evolutionary Consequence of a Trade-Off between Growth and Maintenance along with Ribosomal Damages
Source: PLoS One. 2015 Aug 20;10(8):e0135639. doi: 10.1371/journal.pone.0135639 (PMC4546238; doi:10.1371/journal.pone.0135639)
Supplement: S1 Note — (PDF) [file pone.0135639.s009.pdf]

Supplementary note I

Theoretical analysis based on *r/K* selection

According to the well-known theory of *r*- and *K*-selection, the growth data sets (Fig. 2B, S4 Fig.) were alternatively fitted with the logistic equation (Eq. 1) for the precise evaluation.

$$\frac{dN}{dt} = r(1 - \frac{N}{K})N \qquad \text{Eq. 1}$$

where N, r and K represent the number of cells (cells), the Malthusian growth rate (h^{-1}) and the carrying capacity (cells), respectively. The following solution (Eq. 2.1) was used to estimate the three parameters (K, b, and r), in which K and r were of interest.

$$N(t) = \frac{K}{1 + be^{-rt}} \qquad \text{Eq. 2.1}$$

$$b = e^{-rt_0} \qquad \text{Eq. 2.2}$$

The theoretically estimated values of *r* and *K* are shown in S6 Fig. The Malthusian growth rate decreased and the carrying capacity increased as the starvation and re-growth were repeated, which agreed well with the experimental detection (Fig. 3). If a linear regression of the relations between *r* and *K* is performed, the slope would become steep from R0 to R1 to R7. The results indicated that histidine was primarily used for yield (*K*) in the evolved population R7 rather than for speed (*r*) in the ancestor population R0. Additionally, comparing R0, R1 and R7 at the same nutritional conditions, the trade-off between the growth rate and yield became more significant as the concentration of histidine was reduced. Thus, the repeated starvation and re-growth directed the evolution from *r*- to *K*-selection.

Moreover, the acquired values of *r* were subsequently fitted with the Monod equation (Eq. 3) to evaluate the kinetics of resource utilization (S6 Fig.).

$$r = r_{\max}\frac{S}{K_S + S} \qquad \text{Eq. 3}$$

in which S, r, r_{max} and K_S represent the substrate concentration (histidine, μM), the growth rate (h^{-1}), the maximal growth rate (h^{-1}) and the substrate concentration at which the growth rate is half of r_{max} (μM), respectively. The fitted results showed that both the maximum growth rate and the constant K_S decreased in the order of R0, R1 and R7. Nevertheless, the ratio of r_{max} and K_S, which represents how efficiently the substrate histidine was utilized for cell growth, was increased in the reversed order of R7, R1 and R0. Therefore, the evolutionary transition in growth fitness was coordinated with the improved efficiency of resource utilization, suggesting an evolutionary strategy related to growth economics, dependent on nutritional conditions.
